# Medical consumerism in the UK, from ‘citizen’s challenge’ to the ‘managed consumer’—A symbol without meaning?

**DOI:** 10.1111/hex.13197

**Published:** 2021-01-21

**Authors:** Steve Iliffe, Jill Manthorpe

**Affiliations:** ^1^ Research Department of Primary Care & Population Health University College London London UK; ^2^ King’s College London London UK

**Keywords:** consumerism: national health service, patient involvement, professional authority: quality of care

## Abstract

**Background:**

In Britain's National Health Service (NHS), medical consumerism is disliked by many doctors but managed by NHS leaders. Managed consumers have choices about treatment options, but are expected to help contain costs, improve quality of care, take part in clinical research and advocacy, and increase productivity. There are so many meanings for medical consumerism that it can be categorized, in post‐structuralist terms, as a ‘symbol without meaning’, but meanings are plentiful in the NHS.

**Policy expectations:**

Choices made by discriminating consumers were expected to improve the quality of medical care for all. Extending choice to the many, and not restricting options to the few, would allow gains from choices to accumulate, so that choice would sustain social solidarity. Managed consumerism would in theory, therefore, instil reasonable choices and responsible behaviours in a moralized citizenry, across the nation. The advocates of New Labour's espousal of medical consumerism expected the accumulative effects of customer choices to challenge professional and occupational power, erode the medical model of health and illness, constrain professional judgements, and open the NHS to new ways of working. Almost all their expectations have been thwarted, so far.

**Conclusions:**

Managed consumerism is far from being a meaningless symbol. This discussion paper explores the territory of managed consumerism and suggests realistic ways to make it more effective in shaping the NHS.

**Patient & Public Contribution:**

We developed the arguments in this discussion paper with insights provided by a lay expert (see Acknowledgements) with experience of consumerism in both public sector management and a disease‐related charity.

## 1 BACKGROUND

Doctors as a whole do not like medical consumerism and many academics are sceptical about it. From its early days in the United States, consumerism was ‘an unwelcome thorn in the medical flesh’.^1^ Patient‐centred and latterly person‐centred and personalized care have gained approval from the medical profession, but patients as consumers are not received so positively.^2^ Patient challenges to physician authority—an original definition of medical consumerism—may worsen the relationships between patient and doctor, lead to prolonged and conflictual encounters and reduced patient concordance with treatments.^3^

In the UK, National Health Service (NHS) patients consume services out of necessity (not want) and the state (not themselves but through taxation) funds their care,^4^ as the recent coronavirus pandemic illustrates. According to Downie, NHS patients cannot become real consumers and doctors could not become simple suppliers of goods and services.^5^ The services which consumerism focuses on—maternity services being a prominent example—are seen as an imperfect means to a desired end (healthiness of child and mother), which is simply not a tradeable commodity.^6^ Consumerist arguments that what consumers want will equate with their best interests are hard for medical professionals to accept. High profile if rare instances of such a clash of ethical frameworks occur, for example, when children are ill with functional (psychologically based) physical symptoms; consumerist parents seeking an organic rather than psychosocial diagnosis may make the child's condition worse not better.^7^

In practitioners’ eyes, consumers are often complaining and litigious, but in their own eyes, medical consumers can have many possible faces: chooser, rebel, identity seeker, hedonist/artist, victim, activist and explorer.^8^ In mental health services, consumers may find it even more empowering to see themselves and others as ‘survivors’.^9^

Elsewhere we have sketched out the development of medical consumerism across three generations of policy and practice.^10^ Whilst understanding and mostly agreeing with the above concerns and reservations, we also see advantages in medical consumerism, when it is defined as patient challenge to physician authority.^11^ In our view, medical consumerism is multi‐faceted and is evolving through its encounters with different kinds of health services, producing different generations of consumers and changing definitions of medical consumerism.

Consumerism in health care has been retrospectively identified and seen as emerging in the United States in the first half of the 20th century. Lee,^12^ for example, suggested that there was a strong vein of consumerism in the United States during the 1930s, with consumers advocating universal health insurance. As this first generation developed in the United States in the 1960s, patients began to challenge aspects of professional authority, and consumerism crossed over to the UK. In the second generation, arising in the 1980s, self‐funding consumers sought their health‐related desires, mostly through body enhancement. The third generation was co‐opted into health‐care systems in both the United States and the UK as ‘managed consumerism’,^13^ starting in the 1990s as market mechanisms became the dominant template for health‐care organizations. Managed consumers often have choices but they are also ‘disciplined’, in that they are encouraged to help contain costs, improve quality of care, and take part in clinical research and advocacy, as well as increase health service productivity.

In this paper, we extend our exploration of the third‐generation model, managed consumerism, as it has developed in Britain's NHS and suggest some policy options that may strengthen it as a force for improving the quality of medical care.

## 2 WHAT IS MEANT BY MEDICAL CONSUMERISM?

McDonald et al^14^ argue that the term consumer has limited value in understanding changes in health services; it conceals as much as it illuminates. It seems antithetical to citizenship, its critical approach undermines the pervasive, hegemonic trust between consumers (patients) and providers (professionals) and in the highly emotionally charged environments of health, illness and death it can generate anxiety in the patient rather than reduce it. Ill people may simply not want to be consumers. Reliance on consumerism as a mechanism for improving the quality of health care could be detrimental to the health of non‐consumers, especially in an ageing population with multiple and complex needs.^15^

Powell and colleagues assert that the term consumer is in danger of collapsing into meaninglessness, with multiple and contrasting perspectives on what consumerism is.^16^ A binary model of ‘choice/exit’ versus ‘voice’ oversimplifies medical consumerism, which appears to have many dimensions, including desire for positive, long‐term, respectful clinical relationships which allow free communication of expectations.^17^

O’Hara describes so many meanings for medical consumerism that it can be categorized, in post‐structuralist terms, as a ‘symbol without meaning’.^18^ Raymond Williams, on the other hand, found powerful if sloganistic meanings; consumer behaviour is American, capitalist and bourgeois, a wasteful illusion promoted by producers.^19^ Others have argued that moral criticism of market reforms in health services should call consumerism into question, because consumerism is fundamentally objectionable.^20^ The many forms that medical consumerism may take are summed up in Table 1.

**TABLE 1:** A typology of the roles, types and faces of contemporary medical consumerism

Authors	Roles, types and faces of consumerism
Newman & Vidler 2006	Consumerism as social movement, transformative way of life, expression of capitalist ideology, source of identity
McDonald et al 2007	Consumer as communicator, explorer, identity maker
Powell et al 2010	Types: individual, participatory, advocacy, fiscal, organizational
	Faces: chooser, rebel, identity seeker, hedonist/artist, victim, activist, explorer
O’Hara 2012	Consumerism enforces collective standards, prevents professional abuse, provides expertise for policy makers
Powell & Boden 2012	Consumerism disseminates complementary therapies and non‐medical ways of conceptualizing the body

This typology expresses the heterogeneity of terms, ideas and constructs used in studies of medical consumerism, but in our view, it also reflects a compressed history of consumerism in Britain. It is easy to forget the history of consumerism, or imagine that it did not exist before the NHS was formed, although the opposite is true. The forms of public involvement in medical services before the NHS reflected the forms of the labour movement, with elected worker‐governors on hospital Boards, oversight of general practitioners by friendly societies (mutuals) and elected local government influence over municipal services.^21^ Almost all these forms of public engagement with the fragmented health services of the pre‐Second World War period were swept away by the centrally controlled NHS in 1948,^20^ as part of the foundation of the British Welfare State The institutions that represented the interests and concerns of health service users (patients) had to be re‐invented.

## 3 REBUILDING EXPRESSIONS OF MEDICAL CONSUMERISM (1961‐1979)

In 1961, an editorial commentary in The Lancet commented on the findings of a survey of what the UK public thought about health and welfare services.^22^ The commentary was entitled ‘Patients as consumers: wants and needs’ and was favourable towards consumerism, in that it acknowledged that the public had currently no means of judging medical services and no means of redress against them.

Consumer groups in the UK proliferated in the nineteen sixties and seventies, some being lobby groups or advocates for specific conditions and others claiming to represent the interests of medical consumers as a whole. Examples of the latter were the Patients Association (founded in 1963), Community Health Councils (established in 1974) and the National Association for the Welfare of Children in Hospital (NAWCH) (founded in 1965).^23^ The organizations with a wider remit promoted participatory democracy, encouraging patients to press for more public involvement in the planning and delivery of services.^20^ The managed consumerism built into the 21st century NHS has its roots in the participatory experiments of the sixties and seventies.

## 4 MEDICAL CONSUMERISM AND THE TURN TO THE MARKET (1979‐1997)

The election of Thatcher's government in 1979 initiated a period in which the collectivist Social Democratic model of engaged consumers shaping both their own experiences and the development of the health service itself gave way to a model of consumerism in which proxy consumers operated in a quasi‐market.^20^, ^24^ The introduction of GP fundholding (in which general practitioners (GPs) purchased specialist services for their patients) and also growth of an active general management system tasked with bettering services created these proxy consumers. The quasi‐market came about when an internal market was constructed by the NHS to let general managers and fundholding GPs purchase services for their patients.^25^ Tellingly, the individual consumer relied on others to guide choices about treatments, in a quasi‐market that was carefully managed.

Consumer challenges to professional authority—the first generation version of medical consumerism—were not part of the supposedly radical reform of the NHS. The position of the medical consumer was as weak under the Thatcher/ Major governments (at least in their early years) as it had been under the preceding Labour regimes. This weakness prompted the foundation in 1983 of the College of Health, which aimed to provide information about health and health services, encourage disease prevention, facilitate self‐help and improve relationships between patients and professionals. Various Patients’ Charters and Health Rights guides produced by organizations such as the Consumers’ Association (1983) and the Association of Community Health Councils (1986) prompted the government to publish the Patient's Charter in 1991,^26^ a document that set out essentially symbolic (ie largely unenforceable) rights and standards. The Patient's Charter was widely criticized, but it reflected an attempt by market reformers to take the lead in framing what it meant to be a patient‐consumer in individualized rather than collective terms.^27^

Despite this focus the nearest the Thatcher/Major governments of 1979‐1997 came to promoting medical consumerism in the NHS was through GP fundholding. The consumer in this arrangement was the practice rather than the patient. Fundholding rapidly became controversial and was challenged by Locality Commissioning, which engaged patients in dialogue but concentrated on planning to meet needs within defined communities. Fundholding was wound down after a change in government in 1997 and Locality Commissioning was promoted in its place.

## 5 MEDICAL CONSUMERISM UNDER NEW LABOUR (2000‐2010)

New Labour inherited, therefore, a model of managed consumerism that was emerging from a field in which medical consumerism had many meanings. If medical consumerism has so many meanings, how can we evaluate its impact on health services? One way is exemplified by Newman & Vidler's account of New Labour's adoption of managed consumerism.^28^ In the first years of the New Labour administration (1997‐2000), the government's collectivist approach to NHS reform was clearly different from the Conservatives’ enthusiasm for market mechanisms. From 2000, New Labour adopted a consumerist policy that was designed to reconcile the collectivist Social Democratic conception of the NHS with changes designed to improve ‘middle class buy‐in’ and make private health insurance less ‘necessary’. New Labour's key arguments were as follows: society is being transformed by consumerism and the NHS needs to catch up with the trend; different needs are individually, not socially, distributed; and services should be geared to the interests of users/patients not the convenience of producers or the ‘club culture’ of a hospital, as described by the Kennedy review into Bristol's heart surgery for children.^29^ Using the Gramscian idea of ‘transformation’, Stuart Hall characterized New Labour's efforts to reform the NHS as an example of hybridization, with market mechanisms being the dominant force for change and Social Democratic collectivist practices being the subordinate force.^30^ From the Gramscian perspective, the subordinate force is constantly being transformed into the dominant force, but transformation is itself vulnerable to countervailing power by which subordinates can become dominant. We will return to the possibilities this insight offers, later in this paper.

The mechanisms that were proposed to bring about the shift towards consumerism included the language and imagery of ‘partnership’,^31^ with the patient playing the role of a discriminating but loyal customer. Choices made by discriminating customers were expected to improve the quality of medical care for all, just as wage rises won by trades unionists are received by those not in the union. Extending choice to the many, and not restricting options to the few, would allow gains from choices to accumulate. Thus, choice would sustain social solidarity and allow the vocabulary to expand so that customers could be described as citizens, users, consumers and patients, according to preference.^32^ Extending choice would also give government leverage to support patients against producer dominance and monopoly of knowledge and decision making.

New Labour's plans for reforming the NHS, as distinct from increasing its funding, were ambitious. New Labour governments anticipated that managed consumerism would instil reasonable choices and responsible behaviours in a moralized citizenry, across the nation.^33^ Through alliances that would allow governance of health care for a diverse, differentiated and mobile public, the NHS would appeal to almost all in a political State that expressed, in Gramsci's words, the ‘national‐popular’ character.^34^ This characterization of the NHS as typifying the nation lasted as illustrated by the Olympics opening ceremony of 2012, where the NHS was celebrated as being the best of Britain, and by the Clap for Carers national expression of public gratitude to the NHS during the coronavirus pandemic of 2020.

The advocates of New Labour's espousal of medical consumerism such as Julian Le Grand^35^ and Paul Corrigan,^36^ expected the accumulative effects of discriminating customers to challenge professional and occupational power, erode the medical model of health and illness, constrain professional judgements, and open the NHS to new ways of working. Almost all their expectations have been thwarted, thus far.

## 6 NEW LABOUR’S LIMITED GAINS

In the New Labour period, the NHS did engage with its public in a wider variety of ways, and public and patient representatives did appear in policy meetings, in management of patient‐facing services, and in research projects and programmes. Nonetheless, the changes expected by political advocates of consumerism did not, in the main, appear. The NHS seemed able to minimize consumerist challenges to professional power, and even smooth over tensions between them. Consumerism was reframed around a professionally endorsed aim to involve individual patients in treatment decisions. Consumer choice was welcomed where it extended or amplified a professional ethic. The meaning of ‘choice’ was subtly detached from the political narrative of change and moved towards professionals’ concerns with better models of care.

Newman and Vidler^27^ cautioned against assuming that medical consumerism was a coherent entity to be welcomed or resisted. Instead, they saw it as multi‐faceted, being at least part of the ideology of capitalism, possibly a transformative way of life, conceivably a component of identity or even a social movement (see Table 1). The task, they argued, was to unpack medical consumerism and reveal its actual character and function.

McDonald et al^14^ instead explored the relationship between consumerism and identity. The state's endorsement of consumerism in the NHS promoted a preferred, ideal identity which individuals were encouraged to adopt. Making healthy choices became a sign of the civilized citizen, who thinks of others and of the NHS, as well as of themselves. This consumerist identity implicated people in self‐governance; they had autonomy and individual choice, but the extent of choices depended on being responsible.^37^ This identity, shaped by and for the NHS, is governed and regulated^38^ to promote an ethical self that differs from ‘economic man’ in its altruism. New Labour tried to evoke the co‐operative and mutualist meanings of medical consumerism in the NHS reforms that followed the policy turn towards market mechanisms, in 2000. For example, as Rivett^39^ chronicles:

## 7 SOCIAL DEMOCRACY AND MEDICAL CONSUMERISM

Powell et al^16^ have unpicked the history behind this reframing of medical consumerism. Before the neoliberal turn, consumerism had been a mechanism designed to make collectivist Social Democracy more humanistic and responsive.^17^ Patient groups like those developed by Michael Young (such as the College of Health, Healthline, and also the National Consumer Council) helped to enforce collective standards when the Department of Health often did not know what was actually going on inside the NHS. These consumer groups perceived a need to reform practices across the NHS, from scandals (like experimentation on patients without consent) to failures (the poor care in some long‐stay hospitals). These scandals and failures were (in the consumerist viewpoint) intrinsic to the NHS and required constant vigilance. Consumer groups therefore called for more public participation, to instil voice whilst encouraging loyalty. From the NHS management's perspective, medical consumerism—vocal but loyal—could be used as a way to deepen state knowledge about protection of the vulnerable, and to contribute user expertise to a policy making process dominated by self‐interested policy entrepreneurs from the professions.

Two decades of managed consumerism have revealed the strengths and weaknesses of this Social Democratic engagement with the population. Collective surveillance of governance failings probably has had beneficial effects in most parts of the NHS, but it did not stop the Mid‐Staffordshire Hospital scandal.^40^ Consumerism has probably had more impact on policy than on individual patient experience^17^ but the policies have not necessarily worked in terms of reducing NHS use. For example, NHS Direct, an online information system about health, illness and NHS services aimed at consumers, has had no effect on service use ^8^ and the Expert Patient programme for fostering self‐care of long term conditions may have increased demand on services.^41^ As the coronavirus pandemic has shown, most people using the NHS do not challenge medical authority, and medical discourse, wrapped around intellectual and procedural competence, dominates relationships.

## 8 TAKING A LONG(ER) VIEW

So what should be done about managed consumerism, in the circumstances that we find ourselves in, not the circumstances that we would like? Elsewhere we have argued that consumerism centred on challenges to medical authority will express itself in conflicts over specific decisions in particular people, whilst the excesses of niche market consumerism (typified by ‘cosmetic surgery’) will call for market regulation.^10^ Managed consumerism, on the other hand, may encourage increasing numbers of people to voice opinions that are synoptic as well as individual.

Here realistic expectations need to be fostered. Converting people from being citizens into being consumers—even if it is possible—will not bring about the transformation of the NHS.^30^ The categories of citizen and consumer do not match the identities people seem to have or want. Consumerism as an individual activity is not a mechanism for institutional change; consumers as members of collectivities (localities, Black, Asian and other Minority Ethnic groups, nationalities, sexualities, age, and so on) may be. Future policy should favour expressions of collective managed consumerism.

The regularity with which new strategies to manage consumerism need to be invented suggests that reality tends to be recalcitrant.^31^ Persistent public desires and fears about the trajectory of the NHS suggest that consumerist subjectivity has not been fully installed.^30^ Patient groups were instrumental in launching medical consumerism, but they have largely lost control of the agenda and become subject to management direction. Managed consumerism is not really a symbol without meaning; that is simply a misjudgement attributable to medical consumerism's heterogeneity.

## 9 CONCLUSIONS

Professional hostility to medical consumerism is an understandable but unhelpful response to managed consumerism, which can assist professionals in service development and system reconfiguration. Berwick urges professionals and managers to ‘really listen’ to their publics^42^ and the managed consumers of the NHS can be part of the vocal but loyal response to this imperative. The quality of such listening could become a component of the regulator's (the Care Quality Commission) reviews of NHS organizations.

Managed consumerism is far from being a meaningless symbol, even if it does not have the desired transformational effects on the NHS. This is because it could restore the local involvement in health service management swept away in 1948 whilst also socializing the needs of individual service users or patients. The more patients are engaged with quality of care, decision making and optimal outcomes, the more the dominant consumerist individualism gives way to the formerly subordinate, collective form. In this sense, policy makers’ approaches to public engagement need to be renewed. Managed consumers should be continually engaged in discussions about general policy implementation, as well as their own interests and choices, without unrealistic expectations that managed consumerism will profoundly change the health service.

## 10 AUTHORS’ DISCLAIMERS

The views expressed in this paper are the authors’ alone and should not be interpreted as those of the National Institute for Health Research, the NHS, or the Department of Health and Social Care and its Arm's Length Bodies.

## 11 DISCLAIMER

JM is funded by the National Institute for Health Research (NIHR) Policy Research Unit in Health and Social Care Workforce and the NIHR Applied Research Collaboration South London (NIHR ARC South London) at King's College Hospital NHS Foundation Trust.

## CONFLICT OF INTEREST

None declared.

## Data Availability

Data sharing is not applicable to this article as no data sets were generated or analysed during the current study.

## References

[1] Little S . Consumerism in the doctor‐patient relationship. J Med Ethics. 1981;7:187‐190. 7334494 10.1136/jme.7.4.187 PMC1154953

[2] Gusmano MK , Maschke KJ , Solomon MZ . Patient‐centred care, yes; patients as consumers, no. Health Aff. 2019;38(3):368‐373. 10.1377/hlthaff.2018.05019 30830817

[3] Zeckhauser R , Sommers B . Consumerism in health care: challenges and opportunities. AMA J Ethics. 2013;15(11):988‐992. 10.1001/virtualmentor.2013.15.11.oped1-1311 24257093

[4] Sturgeon D . The business of the NHS: the rise and rise of consumer culture and commodification in the provision of healthcare services. Crit Soc Policy. 2014;34(3):405‐416.

[5] Downie R . Patients and consumers. J Roy Coll Physicians Edinb. 2017;47:261‐265. 29465104 10.4997/JRCPE.2017.311

[6] Leavey R , Wilkin D , Metcalfe DH . Consumerism and general practice. BMJ. 1989;298:737‐739. 2496826 10.1136/bmj.298.6675.737 PMC1836024

[7] Lindley KJ , Glaser D , Milla PJ . Consumerism in healthcare can be detrimental to child health: lessons from children with functional abdominal pain. Arch Dis Child. 2005;90:335‐337. 15781917 10.1136/adc.2003.032524 PMC1720365

[8] Powell JA , Bowden S . Greater choice & control? Health policy in England and the online health consumer. Policy Internet. 2012;4:1‐23.

[9] Tomes N . The patient as a policy factor: a historical case study of the consumer/survivor movement in mental health. Health Aff. 2006;25(3):720‐729s. 10.1377/hlthaff.25.3.720 16684736

[10] Iliffe S , Manthorpe J . Medical consumerism and the modern patient: successful ageing, self‐management and the ‘fantastic prosumer’. J R Soc Med. 2020;113(9):339‐345. 32910877 10.1177/0141076820911574 PMC7488811

[11] Haug M , Lavin B . Consumerism in medicine. Beverly Hills: Sage Publications; 1983.

[12] Lee NS . Framing choice: the origins and impact of consumer rhetoric in US health care debates. Soc Sci Med. 2015;138:136‐143. 26093071 10.1016/j.socscimed.2015.06.007

[13] Bury M , Taylor D . Towards a theory of care transition: from medical dominance to managed consumerism. Soc Theor Health. 2008;6:201‐219.

[14] McDonald R , Mead N , Cheragi‐Sohi S , Bower P , Whalley D , Roland M . Governing the ethical consumer: identity, choice and the primary care medical encounter. Sociol Health Illn. 2007;29(3):430‐456. 17470220 10.1111/j.1467-9566.2007.00493.x

[15] Donaldson C , Lloyd P , Lupton D . Primary health care consumerism amongst elderly Australians. Age Ageing. 1991;20(4):280‐286. 1927736 10.1093/ageing/20.4.280

[16] Powell M , Greener I , Szmigin I , Doheny S , Mills N . Broadening the focus of public service consumerism. Public Manag Rev. 2010;12(3):323‐339.

[17] Song HJ , Dennis S , Levesque J‐F , Harris MF . What do consumers with chronic conditions expect from their interactions with general practitioners? A qualitative study of Australian consumer and provider perspectives. Health Expect. 2020;23(3):707‐716. 32207220 10.1111/hex.13050 PMC7321729

[18] O’Hara G . The complexities of ‘Consumerism’: choice, collectivism and participation within Britain’s National Health Service, c1961‐c.1979. Soc Hist Med. 2012; 26(2):288‐304. 10.1093/shm/hks062 PMC3635502 24771976

[19] Williams R . A vocabulary of culture and society. London, Fontana; 1976: 9.

[20] Sorell T . Morality, consumerism and the internal market in health care. J Med Ethics. 1997;23(2):71‐76. 9134485 10.1136/jme.23.2.71 PMC1377204

[21] Mold A . Patient groups and the construction of the patient‐consumer in Britain: an historical overview. J Soc Pol. 2010;39(4):505‐521. 10.1017/S0047279410000231 PMC2925204 20798768

[22] Anon . Patients as consumers: wants and needs. Lancet. 1961;277:927‐928.

[23] Mold A . Repositioning the patient: patient organizations, consumerism, and autonomy in Britain during the 1960s and 1970s. Bull Hist Med. 2013;87(2):225‐249. 23811711 10.1353/bhm.2013.0022 PMC3752975

[24] Mold A . Making the patient‐consumer in Margaret Thatcher’s Britain. Hist J. 2011;54(2):509‐528. 22826610 10.1017/S0018246X10000646 PMC3401488

[25] Department of Health . Working for patients. London: HMSO; 1989.

[26] Department of Health . The patient’s charter. London: HMSO; 1991.

[27] Mold A . The art of medicine: making British patients into consumers. Lancet. 2015;385:1286‐1287. 25890904 10.1016/S0140-6736(15)60672-9

[28] Newman J , Vidler E . Discriminating customers, responsible patients, empowered users: consumerism and the modernisation of health care. J Soc Policy. 2006;35(2):193‐209.

[29] Kennedy I . Learning from Bristol. London: Stationery Office, CM 5207; 2001.

[30] Hall S . New Labour’s double shuffle. Soundings. 2003;24:10‐24.

[31] Latimer T , Roscamp J , Papanikitas A . Patient‐centredness and consumerism in healthcare – an ideological mess. J R Soc Med. 2017;110(11):425‐427. 28949269 10.1177/0141076817731905 PMC5728618

[32] Clarke J , Newman J . What’s in a name? New Labour’s citizen‐consumers and the re‐making of public services. Paper for the CRESC conference on Culture and social change: disciplinary exchanges Manchester 11th‐13th July 2005.

[33] Clarke J . New Labour’s citizens: activated, empowered, responsibilized, abandoned? Crit Soc Policy. 2005;25(4):447‐463.

[34] Gramsci A . Selections from the Prison Notebooks. London: Lawrence & Wishart; 1971: 131‐134.

[35] Le Grand J . Competition, cooperation, or control? Tales from the British National Health Service. Health Aff. 1999;18(3):27‐39. 10.1377/hlthaff.18.3.27 10388198

[36] Corrigan P . Renewing centre‐left Labour Party politics and policy for the NHS and health. In: Stubbs E , ed. The health of the nation. London: Civitas; 2016.

[37] Warde A . Consumption, identity‐formation and uncertainty. Sociology. 1994;28(4):877‐898.

[38] Barnett C , Cloke P , Clarke N , Malpass A . Consuming ethics: articulating the subjects and spaces of ethical consumption. Antipode. 2005;37(1):23‐45.

[39] Rivett G . The History of the NHS, Chapter 7. Nuffield Trust, London, (no date). https://www.nuffieldtrust.org.uk/chapter/1998‐2007‐labour‐s‐decade

[40] Francis R . Report of the Mid Staffordshire NHS Foundation Trust Public Inquiry. London: House of Commons; 2013. https://assets.publishing.service.gov.uk/government/uploads/system/uploads/attachment_data/file/279124/0947.pdf

[41] Kennedy A , Rogers A , Bower P . Support for self care for patients with chronic disease. BMJ. 2007;335(7627):968‐970. 17991978 10.1136/bmj.39372.540903.94 PMC2071971

[42] Berwick D . Era 3 for medicine & health care. JAMA. 2016;315(13):1329‐1330. 26940610 10.1001/jama.2016.1509

